# Mathematical Modeling of Early Cellular Innate and Adaptive Immune Responses to Ischemia/Reperfusion Injury and Solid Organ Allotransplantation

**DOI:** 10.3389/fimmu.2015.00484

**Published:** 2015-09-25

**Authors:** Judy D. Day, Diana M. Metes, Yoram Vodovotz

**Affiliations:** ^1^Department of Mathematics, University of Tennessee, Knoxville, TN, USA; ^2^National Institute for Mathematical and Biological Synthesis, Knoxville, TN, USA; ^3^Department of Surgery and Immunology, Starzl Transplantation Institute, University of Pittsburgh, Pittsburgh, PA, USA; ^4^Department of Surgery, University of Pittsburgh, Pittsburgh, PA, USA; ^5^Center for Inflammation and Regenerative Modeling, McGowan Institute for Regenerative Medicine, Pittsburgh, PA, USA

**Keywords:** DAMPs, allo-recognition, ischemia/reperfusion injury, transplant, equation-based model, ordinary differential equations

## Abstract

A mathematical model of the early inflammatory response in transplantation is formulated with ordinary differential equations. We first consider the inflammatory events associated only with the initial surgical procedure and the subsequent ischemia/reperfusion (I/R) events that cause tissue damage to the host as well as the donor graft. These events release damage-associated molecular pattern molecules (DAMPs), thereby initiating an acute inflammatory response. In simulations of this model, resolution of inflammation depends on the severity of the tissue damage caused by these events and the patient’s (co)-morbidities. We augment a portion of a previously published mathematical model of acute inflammation with the inflammatory effects of T cells in the absence of antigenic allograft mismatch (but with DAMP release proportional to the degree of graft damage prior to transplant). Finally, we include the antigenic mismatch of the graft, which leads to the stimulation of potent memory T cell responses, leading to further DAMP release from the graft and concomitant increase in allograft damage. Regulatory mechanisms are also included at the final stage. Our simulations suggest that surgical injury and I/R-induced graft damage can be well-tolerated by the recipient when each is present alone, but that their combination (along with antigenic mismatch) may lead to acute rejection, as seen clinically in a subset of patients. An emergent phenomenon from our simulations is that low-level DAMP release can tolerize the recipient to a mismatched allograft, whereas different restimulation regimens resulted in an exaggerated rejection response, in agreement with published studies. We suggest that mechanistic mathematical models might serve as an adjunct for patient- or sub-group-specific predictions, simulated clinical studies, and rational design of immunosuppression.

## Introduction

Solid organ transplantation represents the treatment of choice for end-stage organ failure-associated diseases, and has proved effective at extending and improving the quality of life of patients. Approximately 22,000 patients receive solid organ transplants every year in the United States, according to United Network for Organ Sharing^1^. While 1-year outcomes after solid organ transplantation are excellent, the long-term outcomes are still mediocre, and range from 70% survival rate for kidney transplantation to 40–50% survival for heart/lung and intestine transplantation at 5 years (1–3). These poor long-term outcomes depend on multiple factors related to both donor and recipient, but are in their vast majority dictated by initial polyclonal, multimodal, and redundant innate and adaptive immune responses of the recipient directed against the allograft (4). These early immune responses occur both locally and systemically, in response to non-specific inflammatory damage-associated molecular pattern molecules (DAMPs) or to allo-antigen (allo-Ag)-specific major histocompatibility complex (MHC)-mismatch. These responses may be triggered by (i) the transplant surgery procedure (5); (ii) the type and the quality of the graft, including the level of ischemia/reperfusion (I/R) injury (IRI) post-revascularization; and (iii) the level of pre-formed cellular (T cells) allogeneic and heterologous immunologic memory responses (4, 6).

## Inflammation and Immunity in Solid Organ Transplantation

While most work in the transplant field has focused on the antigen-driven immune processes that drive graft rejection, recent work has begun to focus on the interplay between early innate immune mechanisms and subsequent antigen-driven responses (7–10). In this respect, the transplant community has begun to acknowledge the tightly woven interplay between innate and adaptive immunity that has been recognized in other fields (11–20). These studies have pointed to multiple intersecting pathways by which early stress or injury leads to activation of innate and adaptive lymphoid pathways. Key among these pathways are those driven by DAMPs, which play intracellular housekeeping roles normally but which are released both locally and systemically upon stress, injury, or infection (21, 22). DAMPs activate classical innate immune cells such as macrophages and polymorphonuclear cells (PMN; i.e., neutrophils), but also stimulate dendritic cells (DC) to drive cytotoxic (Tc) and helper (TH) T cell activation/polarization (23–26). In addition, non-conventional γδ-T cells, natural killer (NK)-T cells, as well as TH1 and TH17 cells (along with innate cells) provide other points of intersection between innate and antigen-specific (adaptive) immune responses (6, 27).

The transplantation procedure involves oxygen deprivation (ischemia) in the recipient host tissues as well as in the donor graft due to the time interval from donor organ removal to its placement in the recipient host. Once the transplant is complete, blood flow resumes, a process known as reperfusion. The I/R event is well-known to cause injury (IRI) to tissues, in addition to any direct tissue damage from the surgical procedure. These injurious events further initiate release of DAMPs, and this abates as IRI resolves (28–31). However, DAMPs initiate an acute inflammatory cascade involving the early expression of adhesion and co-stimulation molecules, chemokine release, and the inflammatory cytokine production by innate immune cells as well as memory T cells. Briefly, neutrophils respond to DAMPs by extruding highly inflammatory DNA material [neutrophil extracellular traps (NETs)] that trigger monocytes and tissue macrophages to secrete interleukins (IL-) IL-1β, IL-6, and tumor necrosis factor-α (TNF-α). In turn, these pro-inflammatory cytokines stimulate monocyte-derived DC to produce IL-12, a pivotal cytokine for generation of type-1 immunity (6, 27, 32, 33). In addition, activated monocytes can release IL-23, a cytokine critical for recruitment of IL-17-producing γδ-T cells, responsible in turn for neutrophil chemotaxis and activation (34, 35). As a result of the innate immune cell cytokine storm, the direct response to DAMPs, γδ-T cells, and memory T cells further contribute to IRI by IL-17 and interferon-γ (IFN-γ) release and costimulatory molecule up-regulation in an allo-Ag-independent manner (27, 36, 37).

A second layer of effector and inflammatory molecules is released by pre-formed alloreactive memory Type-1 and Type-17 T cells in response to graft mismatched allo-Ag recognition. The levels of T cell pre-sensitization of the recipient to the donor correlate directly with early acute rejection episodes (38). The ensuing inflammation acts as a feedback loop, and may further cause tissue damage that drives additional release of DAMPs and allo-Ags. Resolution of cellular and tissue inflammation triggered by surgery, IRI, and subsequent DAMP release is mediated by innate regulatory macrophages (M2 and Mreg), intrinsic regulatory cytokines [IL-10, IL-4, and transforming growth factor-β1 (TGF-β1)] along with T regulatory cells (Tregs) in animal models of heart, kidney, and liver transplantation (27, 39–42), while pre-formed alloreactive memory T cells seem less sensitive to regulation by Tregs (43).

These immunologic events may play a significant role in driving the diverse outcomes that accompany organ transplantation in various cases of apparent antigenic mismatch. We use the term “apparent antigenic mismatch” since the response to allo-Ag includes multiple factors, such as (1) actual allo-Ag differences; (2) individual, genetically predetermined thresholds of immune activation in response to a given degree of antigenic mismatch; (3) pre-existing levels of memory T cells; and (4) individual-specific response to immunosuppressive therapy.

Modern organ transplantation has utilized potent strategies to control these unwanted, early immune responses. Specifically, thorough pre-transplant screening of recipient’s pre-formed donor-specific allo-antibody reactivity against the donor (cross-match screening for humoral sensitization) is combined with depleting or non-depleting induction therapy at organ implantation and with versatile maintenance immunosuppression (44–46). All of these methods seek to mitigate the deleterious effects of immunity while allowing regulatory molecules and cells to develop. Notably, these strategies target mostly adaptive immune cells such as T cells, leaving the innate immune players mostly unchecked. Thus, patients with elevated DAMP release and inflammation – due to significant IRI after reperfusion that carry undetected memory T cells to the donor MHC – may experience early rejection episodes despite proper pre-transplant screening, induction therapy, and maintenance immunosuppression. This contrasts with non-sensitized or minimally sensitized patients who experience minimal IRI due to live donation and/or optimal MHC matching, resulting in either indolent subclinical inflammation or in uneventful clinical course with desirable quiescent outcomes. For example, acute cellular rejection (ACR) events in the first 3 months after kidney transplantation occur in 10–12% of patients, while biopsy-proven subclinical rejection occurs in an additional 15–18% of kidney recipients (47).

## Deciphering the Complexity of Inflammation and Immunity with Mathematical Models

The foregoing discussion suggests an emerging paradigm in which context and timing matter more than semantic distinctions among immune/inflammatory responses: in essence, inflammation/innate immunity triggers early memory lymphoid pathways that can subsequently become more focused after exposure to specific antigens, while chronic inflammation might be thought of as the chronic restarting of acute inflammation (48). In this context, attempting to define and predict responses under particular circumstances, especially in individuals, becomes almost overwhelmingly complex.

Mathematical modeling provides a key tool by which to study the integrated innate/adaptive response or acute/chronic inflammatory response and thereby untangle some of this complexity (48–50). Therefore, such models provide a means to drive novel hypotheses with regard to complex immune processes like those involved in the transplantation procedure and can assist in identifying viable – and possible novel – points of control or diagnostic biomarkers. Multiple mathematical models that integrate innate and adaptive immune responses have been developed over the past decade to address diverse questions and disease states (51–54). However, a comprehensive mathematical model of organ transplantation is as yet lacking, and the complexity of the immune events involved in the procedure reiterates the need for such an approach. Complex systems, especially biological ones, are notoriously sensitive to initial conditions (55, 56). Thus, to address the solid organ transplant process comprehensively, we hypothesize the need to model not only the transplant and its antigenic properties, but also the initial conditions relating to the transplant surgery and subsequent IRI as drivers of innate immunity. Indeed, prior mathematical modeling studies have suggested the need to model the underlying process, for example, in the case of the role of underlying trauma in the setting of hemorrhagic shock (57).

The modeling simulations in this present study suggest that surgical injury and graft damage can be well-tolerated by the recipient when each is present alone, but that their combination (along with antigenic mismatch) may lead to acute rejection. An emergent phenomenon from our simulations is that low-level DAMP release can tolerize the recipient to a mismatched graft under specific restimulation settings, while other restimulation regimens lead to an exaggerated rejection response.

## Results

To examine the early stages of inflammatory/immune responses to an organ transplant, including investigating the role of IRI in transplantation, we developed a mathematical model that includes the inflammatory hallmarks of IRI as well as the immune responses elicited by the apparent antigenic mismatch of the graft. As described above, we use the term “apparent antigenic mismatch” to comprise (1) actual antigenic differences; (2) individual, genetically predetermined thresholds of immune activation in response to a given degree of antigenic mismatch; (3) pre-existing levels of memory T cells; and (4) individual-specific response to immunosuppressive therapy.

The degree of this apparent antigenic mismatch is governed by a parameter, α, wherein a value of zero implies that the graft has 0% apparent mismatch with the host and a value of 1 implies complete (i.e., 100%) apparent mismatch. The model is initiated with a specified level of initial damage to the host and to the graft from the surgery and I/R, and thus the model simulations begin at approximately the time that transplant surgery is concluded (~8 h after the surgery begins), at which time reperfusion would occur.

In order to increase our ability to analyze qualitatively the driving forces behind diverse transplant outcomes, we simplify the number of components considered in the model and aim to create an abstract representation of the processes mentioned above. We focus on the following core scenarios and outcomes:

1. Clinical quiescence: the graft, following transplantation, shows no signs of inflammatory infiltrates. This is represented by model simulations showing little or no graft damage and corresponding to fully or almost fully recovered graft functionality.
2. Acute clinical rejection: the graft, following transplantation, sustains levels of damage from the host response that cause it to lose functionality, occurring in the first 3 months after transplant. This is represented by model simulations showing high graft damage and corresponding poor graft functionality very early after the simulation is initiated (i.e., after the transplant is completed).
3. Subclinical inflammation: the allograft, following transplantation, shows no apparent clinical signs of organ damage, but subclinical levels of inflammation and cellular infiltrates are detected in the protocol biopsies in the first 3 months after surgery. This is represented by model simulations showing either stabilized but diminished graft functionality due to lingering inflammation, or non-stabilized, poor graft functionality due to oscillating inflammatory responses driven by T cells.

### The mathematical model

**Figure 1:**
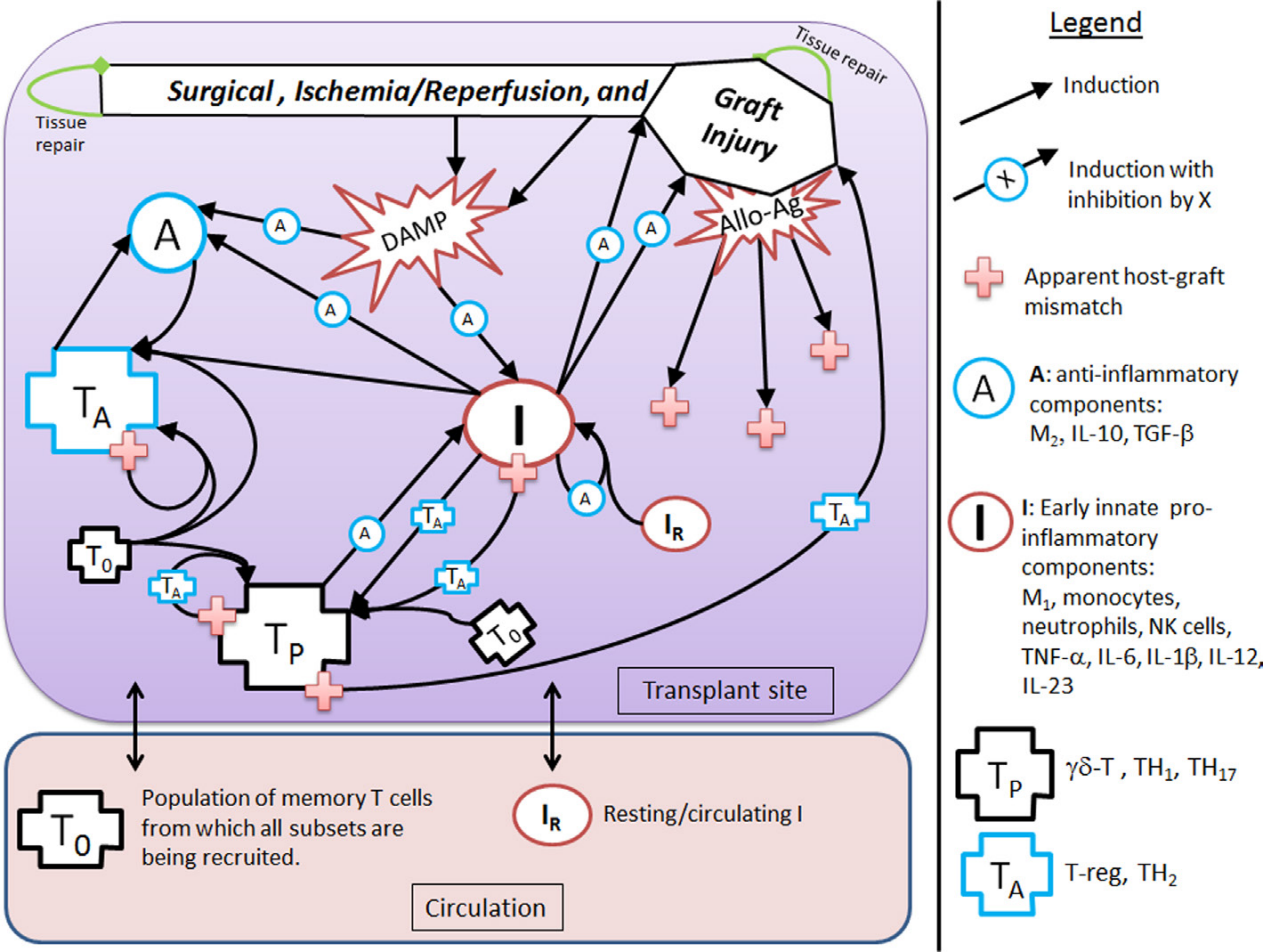
**Interaction diagram**. The diagram provides an abstract, high-level view of the immune and inflammatory processes involved in solid organ transplant that we include in our mathematical model. Four dynamic immune variables are defined: *I*, *A*, *T*_P_, and *T*_A_ as described in the figure legend next to their respective graphic marker. Also tracked is host tissue damage and graft tissue damage via the dynamic variables, *D* and *D*_G_, which are represented in the diagram by the shape labeled “Surgical, Ischemia/Reperfusion, and Graft Injury” at the top of the diagram, along with DAMP release as a result of this injury. Arrows represent induction/activation of a target variable (connected at the arrow head) by an initiating variable (connected at the arrow tail). Inhibitory effects are indicated by the presence of an inhibitory variable marker resting atop the middle part of an arrow. For example, *A* inhibits the activation of *I* from DAMPs released by tissue damage. Multiple arrows coalescing into a target variable at the same point indicate that all initiating variables are required to complete that particular induction/activation process. For instance, *I* and *A* are both needed to activate *T*_A_. Circulating/resting source populations of T cells and innate immune components, *T*_0_ and *I*_R_, respectively, are required for all processes that induce/activate these into the variables *T*_P_ or *T*_A_ and *I*, respectively. To keep the diagram uncluttered, the source populations are not shown in all of the processes in which they are required. Instead a representative example is given for each, as seen in the activation of *I*_R_ into *I* by *T*_P_ and in the activation of *T*_0_ into *T*_P_ (alternatively, into *T*_A_) by *T*_P_ (alternatively, by *T*_A_). The presence of allo-Ag of the graft is indicated with a red cross and represents another excitatory factor of the pro-inflammatory arms of the system as is the DAMP release by damaged tissue. Some activation processes require the presence of allo-Ag and these are represented by a red cross at the initiating (tail) end of an arrow.

Figure 1 provides a schematic of all the components and interactions included in the model equations. Table 1 provides a description of the dynamic model variables and Table 3 in the Section “Materials and Methods” explains the auxiliary model variables. The dynamic model variables are those whose rates change over time and are modeled with an ordinary differential equation (ODE); whereas auxiliary variables are functions of dynamic variables. We first discuss the interactions that are pro-inflammatory and then discuss how these processes initiate and/or are inhibited by the anti-inflammatory components, all based on the immunology discussed in Section “Inflammation and Immunity in Solid Organ Transplantation.” The model does not currently take into consideration explicitly the immunosuppressive therapies given before/during the transplantation procedure, though the effect of immunosuppression is in a sense contained in the concept of apparent antigenic mismatch. We envision testing specific immunosuppression mechanisms (e.g., killing of all inflammatory cells vs. specific killing of T cells) in future iterations of this model.

**Table 1:** **Dynamic model variables**.

Dynamic model variable name	Description	Initial condition(s)
*D*	Tissue damage to recipient host; measured in arbitrary units: *D*-units	*D*(0) = *D*_0_ ≥ 0 due to surgery and ischemia reperfusion injury of host
*D*_G_	Graft tissue damage; measured in arbitrary units: *D*_G_-units	*D*_G_(0) = *D*_G0_ ≥ 0 due to ischemia reperfusion injury of graft
*I*	Early innate pro-inflammatory components, such as tissue M1 macrophages, monocytes, neutrophils, TNF-α, and natural killer (NK) cells; measured in arbitrary units: *I*-units	*I*(0) = *I*_0_ = 0 in all of the scenarios considered
*A*	Anti-inflammatory mediators such as IL-10 and TGF-β1; measured in arbitrary units: *A*-units	*A*(0) = *A*_0_ = 0.125 maintains a background level at homeostasis (69)
*T*_P_	Pro-inflammatory T cells such as γδ-T cells, TH1 cells, and TH17 cells; measured in arbitrary units: *T*_P_-units	*T*_P_(0) = T_P0_ = 0
*T*_A_	Anti-inflammatory T cells such as TH2 and regulatory T cells; measured in arbitrary units: *T*_A_-units	*T*_A_(0) = *T*_A0_ = 0

The goal of this modeling exercise is to understand the dynamics of the transplant procedure from a more abstract perspective, in which we group multiple components into a single variable. While this level of abstraction will in no way allow a quantitative prediction of specific mediators and cells, this approach does allow for an examination of the overall qualitative dynamics of this system in which excitatory and inhibitory mechanisms interact. The early innate components of the model, denoted by the variable *I*, incorporate the general pro-inflammatory effects of cells such as tissue-resident M1 macrophages, circulating monocytes, neutrophils, and NK cells as well as cytokines such as TNF-α, IL-6, IL-1β, IL-12, and IL-23. Pro-inflammatory T cells are represented by the variable *T*_P_, and incorporate the general properties of γδ-T cells, TH1, and TH17 T cell subsets. Also included are anti-inflammatory components, denoted by *A*, which include M2 macrophages, IL-10, and TGF-β1. In addition, anti-inflammatory T cells are denoted by the variable *T*_A_ and are comprised of T regulatory and TH2 T cells. There are also two dynamic variables that track the rate of change of tissue damage: one for host tissue, denoted by the variable *D*, and another for graft tissue, denoted by the variable *D*_G_. These six dynamic variables are modeled with ODEs that describe how the rates of these entities change over time as they interact with one another under different simulation scenarios. The variables have arbitrary units, as we are not aiming to match them with quantitative data but instead examine their dynamic behavior. The time scale is in hours. Whereas some parameters governing the various rates of the interactions are estimated from the literature when possible (e.g., from half-lives of cells and inflammatory mediators), the parameters are largely estimated to constrain the model to display basic biologically feasible behavior; see Section “Materials and Methods” for more information.

Figure 1 shows that *D* and *I* interact in a positive feedback loop that is inhibited by *A*. This models the effect of DAMPs released by tissue damaged due to IRI. This process is driven by early innate immune components, resulting in the activation of pro-inflammatory components from a resting/circulating population, *I*_R_ (5). These activated pro-inflammatory components cause further tissue damage; but the activation is inhibited by anti-inflammatory influences in a “checks-and-balances” manner. However, severe damage can cause an unabated positive feedback loop among these components, resulting in an unresolved response (31, 57). In the absence of graft placement (i.e., considering the surgical procedure alone), the innate pro-inflammatory components can also induce pro-inflammatory memory T cell recruitment from a circulating T cell population, *T*_0_ (9, 58). In the presence of the anti-inflammatory components, *A*, the innate components, *I*, can induce Tregs and TH2 cells, represented by *T*_A_. Many of these activation/induction processes are inhibited by either *A* or *T*_A_ (27, 41, 59–61). This describes the interactions surrounding the surgical procedure and IRI of the host.

When a solid organ is transplanted, we considered that it would have some initial IRI due to the removal and transport procedures. In addition, the organ could subsequently be damaged by the pro-inflammatory components (both innate and T cell-mediated) present at the transplant site, even in the absence of allo-Ag (58). We model graft functionality (percent), *G*, as a function of this damage, *D*_G_ (see Table 3 in “Materials and Methods”). Subsequently, we include a parameter (α) governing the mismatch factor to scale the response from innate and T cell pro-inflammatory components in response to an allograft. Figure 1 also shows that graft injury can release DAMPs, which in turn can activate innate immune components as discussed above. Furthermore, the presence of a graft with a positive antigenic mismatch factor, governed by the parameter α, will cause antigen-specific memory T cells to infiltrate and cause further injury to the graft. This process is modeled by a gain to *D*_G_. This damage will reduce graft function, *G*, as illustrated in the inset figure of Table 3, and consequently will reduce the percentage of graft tissue available to harm further.

With a positive graft mismatch factor, the early innate pro-inflammatory components, such as monocytes and M1 macrophages, through allo-recognition, will provide additional and specific activation via DC of the pro-inflammatory memory T cells, *T*_P_ (10, 58). This process is indicated in Figure 1 by the arrow coming from *I* into *T*_P_, with the apparent host–graft mismatch marker (red plus sign) present at the tail end of the arrow. In keeping with the abstract model representation of these processes, we do not include the DC component directly, yet the process is implicit in the interactions. Additionally, a positive graft mismatch factor will enhance further recruitment/activation of both pro- and anti-inflammatory T cells, from the source T cell population, *T*_0_, by already activated components of these types. Again, various processes are inhibited by *A* and/or *T*_A_, as indicated in the legend of Figure 1 by an induction arrow that has a particular variable marker sitting atop it in the middle.

**Figure 2:**
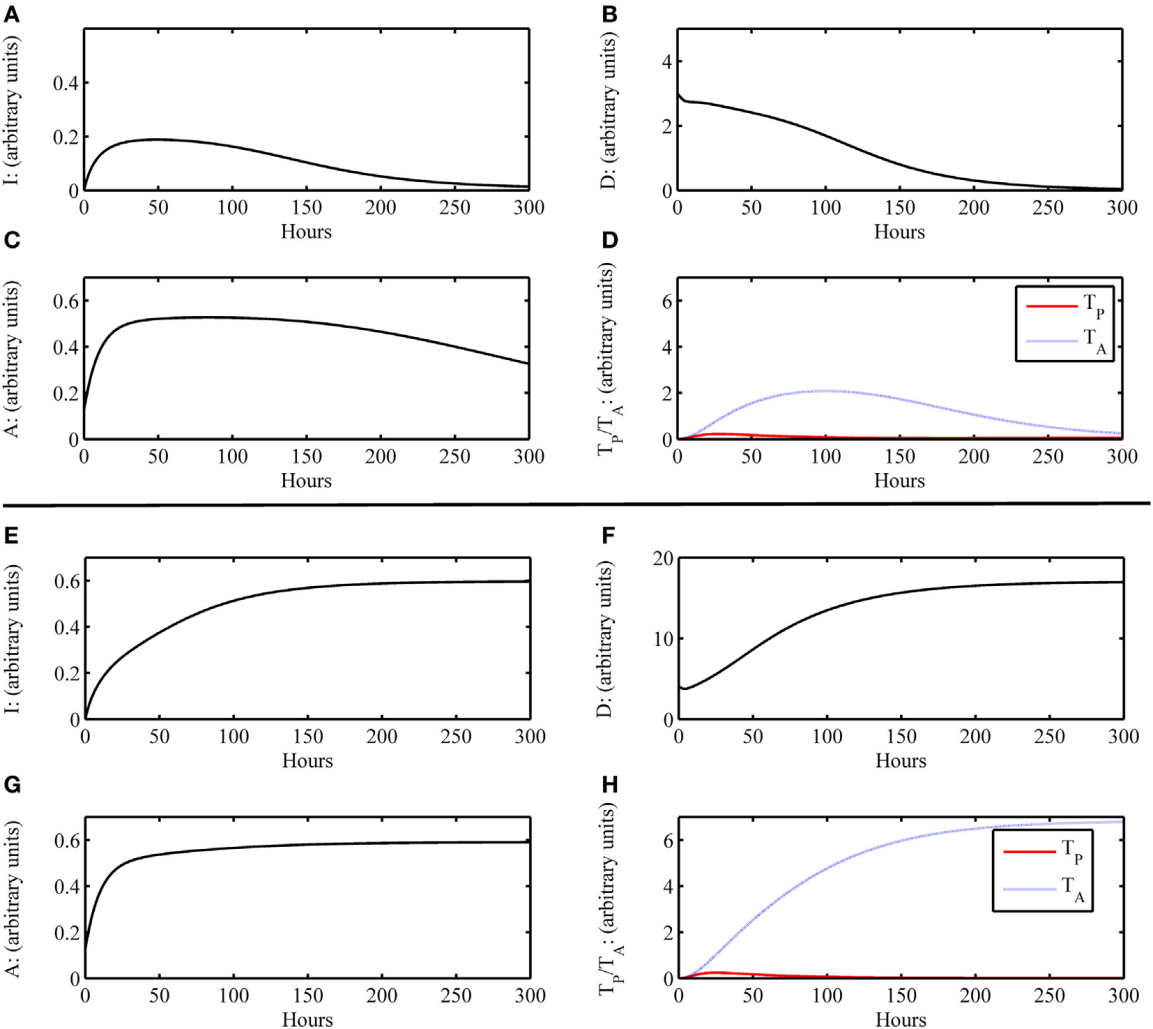
**Simulation results of the inflammatory cascade following transplant surgery only without graft placement (i.e., *G* = 0)**. **(A–D)** Below a certain threshold, initial host tissue damage caused by IRI incites an inflammatory response that resolves to baseline levels. Initial condition for this simulation was (*I*_0_*, D*_0_*, A*_0_*, D*_G0_*, T*_P0_*, T*_A0)_ = (0, 3, 0, 0.125, 0, 0) with parameters as given in Table 4. For *D* < 4, this outcome is possible. **(E–H)** Above a certain threshold, initial host tissue damage caused by IRI incites an inflammatory response that does not resolve and results in host health failure. Note that this scenario is not the one we would consider for transplant conditions, but demonstrate the scope of the model dynamics to produce theoretically possible outcomes of traumatic injury. Initial condition for this simulation was (*I*_0_*, D*_0_*, A*_0_*, D*_G0_*, T*_P0_*, T*_A0_) = (0, 4, 0, 0.125, 0, 0) with parameters as given in Table 4. For *D* ≥ 4, this outcome is possible.

In the Section “Materials and Methods,” the construction of the model is discussed and the full model is given by Eqs 1–6, with the model parameter descriptions and values used in the simulations given in Table 4. The equations are solved numerically to produce time courses of each of the system variables or states (see Materials and Methods). These resulting time courses are translated to clinical outcomes in the following manner. In general, we define a pre-surgery initial condition for the model variables as (*I*_0_*, D*_0_*, A*_0_*, D*_G0_*, T*_P0_*, T*_A0_) = (0, 0, 0.125, 0, 0, 0), which indicate that all system components are at their background values. This state is referred to as the baseline equilibrium. This setting assumes that there are no underlying immune conditions prior to transplant surgery, which is typically not realistic in the case of transplant recipients. Future iterations of the model could incorporate prior host health conditions. The system can be perturbed from this baseline state, for instance, by setting a non-zero initial condition for *D* and/or *D*_G_, which indicates the presence of damaged tissue to host and/or graft, respectively, due to IRI. The rates at which system variables change as a function of time are governed by the Eqs 1–6. A simulation in which the variables’ time courses return to the background levels, after a brief transient increase away from this state due to perturbation, is translated as a healthy outcome. Figures 2A–D display a basic healthy outcome scenario in terms of host health.

**Figure 3:**
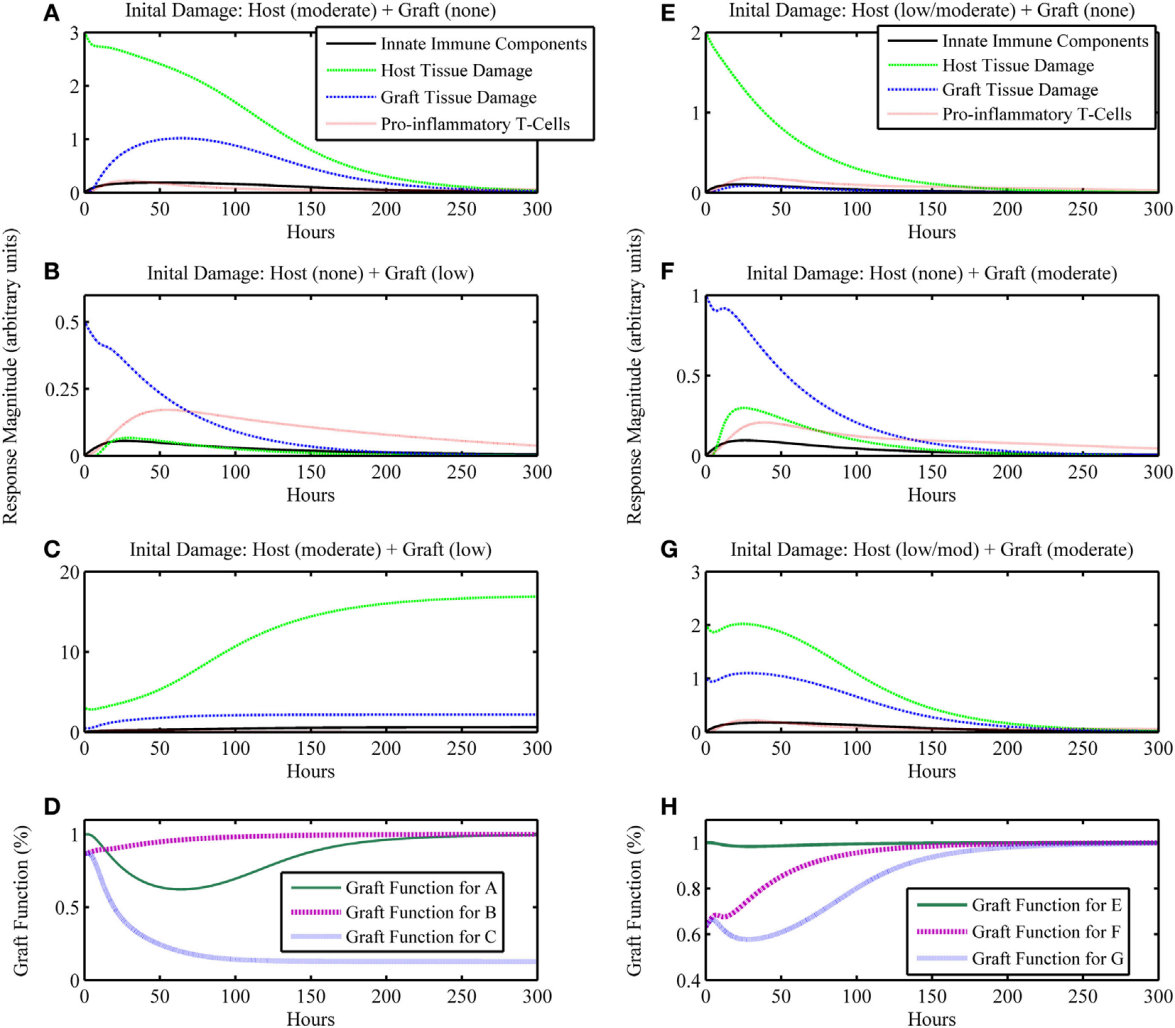
**Simulation results of the inflammatory cascade following transplant surgery and non-allo-Ag graft placement (i.e., α = 0)**. Combined initial host and graft IRI can synergize to incite an inflammatory response that **(A–D)** cannot resolve, causing graft failure or **(E–H)** transiently decrease graft function significantly. **(A–C)** present a series of simulations in which **(A)** a moderate level of initial surgical IRI in the host is considered with no corresponding graft IRI associated with the placement, **(B)** no initial surgical IRI in the host is considered with a low level of initial graft IRI, or **(C)** the moderate level of initial surgical IRI in the host of simulation **(A)** is coupled with the low level of initial graft IRI of simulation **(B)**. In **(D)**, the graft functionality curves corresponding to simulations **(A–C)** are shown. The “Graft function for C” time course in **(D)** displays the synergy to severely affect graft function such that the graft fails, shown as functionality decreasing to and remaining at 12%. Similarly, panels **(E–G)** display outcomes for **(E)** a low/moderate level of initial surgical IRI in the host with no corresponding graft IRI associated with the placement, **(F)** no initial surgical IRI in the host with a corresponding moderate level of initial graft IRI, or **(G)** the combination of the low/moderate initial level of surgical IRI in the host from simulation **(E)** with the moderate level of initial graft IRI from simulation **(F)**. In **(H)**, the graft functionality curves corresponding with **(E–G)** are shown. The “Graft function for G” time course in **(H)** displays the synergy to significantly affect graft function, but only transiently after which the graft functionality fully recovers. Initial conditions for **(C)**: (*I*_0_*, D*_0_*, A*_0_*, D*_G0_*, T*_P0_*, T*_A0_) = (0, 3, 0.5, 0.125, 0, 0); initial conditions for **(G)**: (*I*_0_*, D*_0_*, A*_0_*, D*_G0_*, T*_P0_*, T*_A0_) = (0, 2, 1, 0.125, 0, 0).

On the other hand, an unhealthy outcome is presumed if the departure away from the healthy equilibrium is not transient but instead causes the variables to approach a different equilibrium that has elevated levels of the variable states. The unhealthy equilibrium implies host health failure and, when a graft is considered, graft failure as well. Alternatively, one could define a level of cumulative damage that could be considered as irreparable, rather than defining non-recovery only by the system’s long-term behavior; we did not explore this possibility in the present study. Figures 2E–H display a basic unhealthy outcome scenario in terms of host health. When a graft placement is considered (with and without apparent mismatch), outcomes also include the percent graft functionality, where a steady-state graft functionality value of 12% represents outright graft failure. See Figures 3A–D, for instance.

### Simulation: Ischemia/reperfusion injury without graft placement (i.e., *G* = 0)

As a first scenario, we consider only the aspects of the inflammatory response of the host involved during the surgical transplant procedure in the absence of a graft placement. This scenario could also be viewed as a look at the trauma of transplant surgery or an instance of accidental blunt trauma, in general. To simulate this situation, we set the initial condition for the host damage variable, *D*, to a non-zero value, and remove the presence of the graft, *G*, from the model. All other variable initial conditions are set to their healthy state baseline values. Figures 2A–D show that an inflammatory response is incited (e.g., levels of *I*, etc. increase from baseline values) for some initial level of host tissue injury corresponding to DAMP release. We note that this response resolves completely in a reasonable time frame. In other words, the inherent inhibitory mechanisms provided by the anti-inflammatory variables, *A* and *T*_A_, are sufficient to regulate the response correctly. However, in Figures 2E–H, the initial level of IRI was high enough to cause irreparable damage, an unlikely situation in today’s modern operating theater, yet a theoretically possible outcome. Thus, our model displays feasible qualitative behavior related to surgical trauma.

### Simulation: Ischemia/reperfusion injury with graft placement but with no apparent antigenic mismatch (i.e., α = 0)

The next iteration of simulations considers not only the IRI to the host from surgery but also the IRI associated with the graft due to the processes of harvest from donor and transportation to the recipient host. We assume that initial graft functionality starting at a percentage lower than 100% is a result of IRI due to the harvest and transport procedures, and not an indicator of the functionality that it had when still intact in the host from whom the graft was harvested. Thus, 100% in our model would mean 100% of the total functionality exhibited by a given organ pre-transplant. Presumably, organs harvested for transplant were functioning “normally,” such that they did not have existing damage affecting this *normal* function. However, this value could be lower if an organ were harvested from an older or less healthy donor (a scenario we did not explore explicitly). For this simulation set, we assume that the graft and host are identical, and therefore do not consider any interactions that involve allo-recognition due to mismatch (i.e., the parameter governing mismatch intensity is set to zero: α = 0). The model also displays feasible qualitative behavior for possible outcomes when considering ranges of injury severity. In Figures 3A–D, we show that initial host damage combined with initial graft damage can synergize to result in graft failure, whereas each of these challenges separately did not. Figures 3E–H show synergy as well, but in a less extreme manner, wherein the graft does not fail and recovers fully. However, as seen in Figure 3H, the time course for “Graft Function for G” shows that the negative effects on graft function from IRI reduce graft function by 60% at one point in the simulation. This result suggests that the non-specific, detrimental effects of inflammatory processes initiated by IRI may make the graft that much more vulnerable in cases where host–graft mismatch is considered. We explore mismatch scenarios in the next two sections.

### Simulation: Ischemia reperfusion injury with graft placement and varying apparent antigenic mismatch levels (i.e., α > 0)

**Figure 4:**
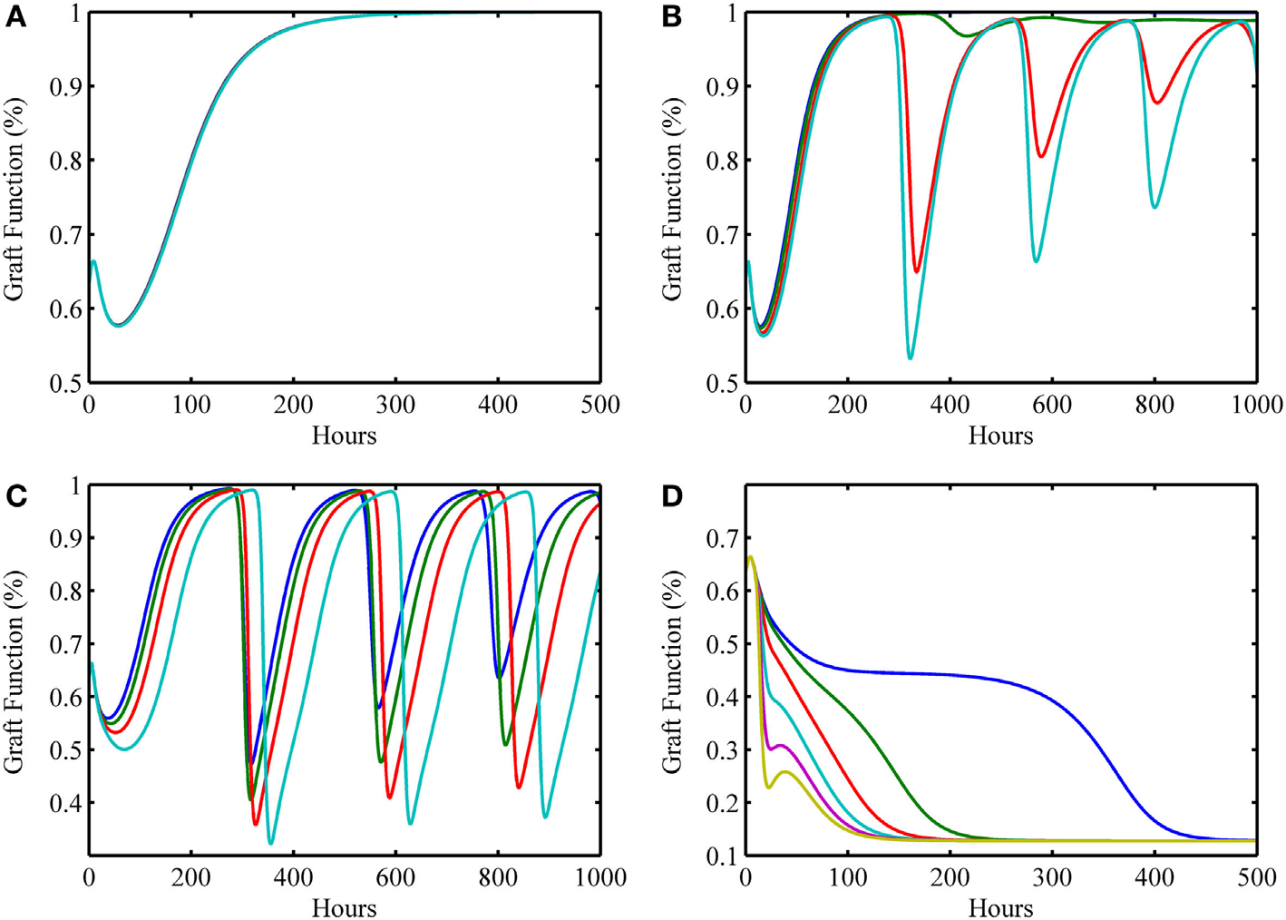
**Simulation results showing outcomes of transplant surgery with placement of allo-Ag graft for various degrees of apparent mismatch (i.e., α > 0)**. The initial condition used in Figure 3G [i.e., (*I*_0_*, D*_0_*, A*_0_*, D*_G0_*, T*_P0_*, T*_A0_) = (0, 2, 1, 0.125, 0, 0)] was also used here but now various values of the apparent mismatch factor parameter, α, were explored to observe the effects of initial host and graft tissue damage from IRI in conjunction with allo-recognition. **(A)** With low mismatch factor (α = 0–0.03), graft tolerance is seen. **(B)** Within a higher range (α = 0.04–0.25), damped oscillations in graft functionality appear but resolve to greater than 95% functionality in the long term with values of α on the higher end of the range taking months to resolve and stabilize. **(C)** Within the next highest interval (α = 0.29–0.5), undamped oscillations are apparent. This indicates a regime where graft function is affected by chronic inflammation driven by T cells that flares up and subsides periodically. **(D)** The last interval (α = 0.55–1.0) displays acute graft failure within 400 h for α values near the minimum of this range and within 125 h near the maximum of this range.

In this next simulation set, we consider varying levels of host–graft mismatch, and thus the interactions shown in Figure 1 involving allo-recognition come into play. We use the initial condition (*I*_0_*, D*_0_*, A*_0_*, D*_G0_*, T*_P0_*, T*_A0_) = (0, 2, 1, 0.125, 0, 0) as in Figure 3G, and set α to different values within the interval [0,1] in the multiple simulation runs. Figures 4A–D display four qualitatively different outcome scenarios corresponding to ranges of the mismatch parameter, α. Each figure panel displays the graft functionality results of multiple simulation runs for values of α within the specified ranges. In these various scenarios, we observe outcomes corresponding to the clinical scenarios mentioned earlier at the beginning of Section “Results.” Clinical quiescence is represented in Figure 4A, where there is little or no graft damage and full or nearly full graft functionality is achieved and retained. Acute clinical rejection is represented in Figure 4D, where poor graft functionality is seen very early after the simulation is initiated (i.e., after the transplant is completed), and failure is predicted to occur within less than a month’s time. The subclinical inflammation outcome is represented in Figures 4B,C. In Figure 4B, we interpret the smaller oscillations as *subclinical chronic* inflammation predicted to resolve on the order of 1–3 months (shown for up to 1000 h ~ 42 days), since the recovery behavior is different from, and takes longer than, the graft tolerance recovery scenario of Figure 4A. Furthermore, since in Figure 4B the damped oscillations are such that (1) graft health does not decrease too often nor too greatly below the original graft health level; and (2) an acceptable recovery is seen eventually (i.e., graft health is greater than 95%), we interpret this behavior as *subclinical*. In other words, the graft is in comparable or better condition than when it was first transplanted, but it is not maintaining optimal function until much later. Note that Figure 4A could also be classified as *subclinical*, but the length of time in which graft health is not ideal is much shorter relative to the scenarios in Figure 4B. Thus, we do not classify Figure 4A as a *chronic* scenario. In Figure 4C, the oscillations are larger and do not resolve as in Figure 4B. We equate this outcome with long-term rejection since a high and steady level of graft function is never observed as T cells cause inflammation and subsequent damage to flare up and subside repeatedly. This prediction points to a scenario leading to graft failure, even though there are times when there is only *subclinical* inflammation, and a good level of graft function is observed.

Table 2 displays a summary of minimal initial graft functionality percentages (corresponding to an initial value of *D*_G_) from which outright graft failure (i.e., ending graft functionality of 12%) is avoidable, given a particular value of α. For ~0.032 < α < ~0.3, the healthy stable equilibrium is replaced by a suboptimal healthy stable equilibrium. Higher α values outside this range give rise to oscillations that indicate worsening graft function, with the minimal graft functionality of the oscillatory range reaching 27% as α approaches 0.7. For α > ~0.75, outright graft failure is the only outcome and the ending graft functionality equilibrium value is 12%.

**Table 2:** **Minimal initial graft function required for graft survival given a particular mismatch intensity factor**.

Value of α	Minimal graft functionality percent [or *D*_G_(0) value]	Ending graft functionality percentage (steady state value of *G*)
0	20% (or 1.9)	100%
0.1	20% (or 1.9)	99%
0.2	20% (or 1.9)	97%
0.3	20% (or 1.9)	75–97% oscillation range
0.4	20% (or 1.9)	48–99% oscillation range
0.5–0.6	20% (or 1.9)	38–99% oscillation range
0.7	24% (or 1.8)	27–99% oscillation range
0.8–1.0	No cutoff exists	Graft failure (13%)

Ending graft functionality percentages are with respect to an initial assumed 100% functionality that a given organ had pre-transplant, as explained in Section “Simulation: Ischemia/Reperfusion Injury with Graft Placement But with No Apparent Antigenic Mismatch (i.e., α = 0).”

### Simulations of preconditioning scenarios

**Figure 5:**
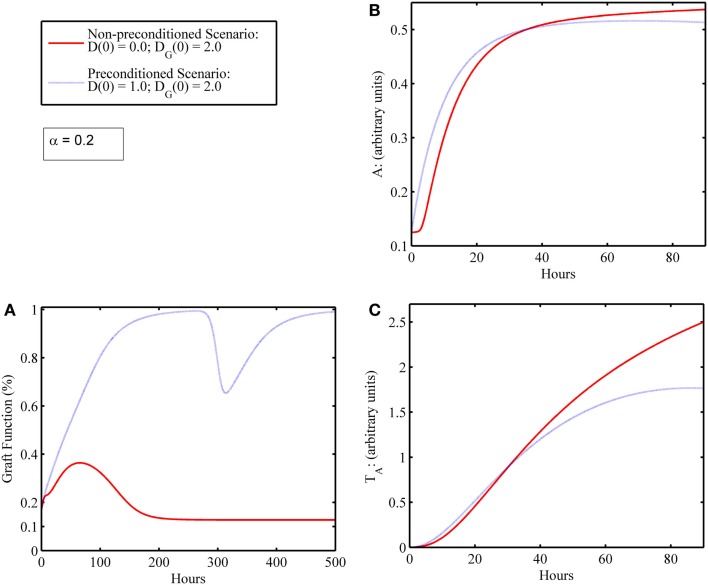
**Preconditioning phenomena: initial surgical IRI allows damaged graft to recover compared to scenario wherein graft failure occurs in the absence of initial surgical damage**. **(A)** Graft functionality with (blue) and without (red) an initial level of host I/R damage, *D*(0). Initial graft damage [*D*_G_(0) = 2] along with a low initial level of host tissue damage [*D*(0) = 1] results in graft recovery to full functionality (blue); whereas initial graft damage [*D_G_*(0) = 2] without the low initial level of host damage [*D*(0) = 0] leads to graft failure (red). **(B)** The anti-inflammatory components, *A*, and **(C)** anti-inflammatory T cells, *T*_A_, with (blue) and without (red) an initial level of host IRI, *D*(0). Comparing the red and blue time courses for both anti-inflammatory variables (*A* and *T*_A_) in **(B,C)**, one observes a slight increase in levels (blue above red) in the first 24 h or so. This increase in the anti-inflammatory variables (especially of *A*) induced by the very inflammatory cascade that was due to DAMP release actually allows for graft survival.

In some simulations, an initial level of host tissue damage can act as a preconditioning factor in promoting graft survival. While the release of DAMPs from injured tissue incites pro-inflammatory components, the cascade also involves induction of anti-inflammatory mediators. If the pro-inflammatory levels from this initial surgical DAMP release are below some threshold, and the corresponding anti-inflammatory cell/mediator levels are above some threshold at the time the additional DAMP release happens from an IR-injured graft, then an attenuated damage response may be possible. We depict one such simulation experiment of this preconditioning phenomenon, shown in Figure 5. This type of preconditioning, in which the response to a second insult is lower than that for the first, is called “tolerance” and has been reported widely in multiple settings of acute inflammation (62, 63). Indeed, a similar tolerance phenomenon was reproduced in a mathematical model of the host immune response to repeated endotoxin challenge (64). That study also demonstrated that repeated endotoxin challenges that were not timed carefully displayed potentiation of the inflammatory response, another manifestation of preconditioning typically known as priming (65). The analogous potentiation feature was seen in the present model in Figure 3D even with no mismatch factor present. We interpret this outcome to be similar to the scenario in which a graft is rejected, and the patient undergoes repeat transplantation. The outcomes in this setting are known to be poor (44, 66). Thus, the timing of the excitatory and inhibitory mechanisms involved in the entire transplant process is important to understand in order for therapeutic strategies to positively synergize with these events.

## Discussion

The integrated nature of inflammatory and antigen-specific immunity that underlie the response to organ transplantation has largely defied a synthetic understanding. This complexity can often be observed in the form of emergent phenomena that cannot be predicted based on an understanding of the component parts of the immune system, and may be at the root of the need for life-long immunosuppression post-transplantation. We suggest that the development of novel treatment strategies for organ transplantation can be aided greatly by mechanistic mathematical models such as the one presented here, because inevitably, independent mechanisms must be integrated in order to predict higher-order system properties in a clinically relevant manner. We regard a mechanistic model as one that describes “rules” for how the individual model components interact and evolve with time. We use the term “mechanistic” to distinguish this type of model from statistical or data-driven models, in which quantitative associations are defined, rather than abstracted mechanisms.

The past decade has witnessed such a synthesis in the form of simplified (reduced-order) computational models of acute inflammation, which have yielded useful insights into the mechanisms and pathophysiology of critical illness (64, 67–69). However, such models are at best only capable of general, high-level predictions, which are not sufficiently specific so as to be testable in individual patients or in *in vitro*/*in vivo* experiments. Alternatively, modeling biological systems in a realistic fashion often necessitates complex, large-scale models describing the underlying system dynamics (54, 70, 71). An important advantage of such mechanistic models is that they can allow for quantitative predictions (48, 49, 56, 72–76) and clinically translational connections of molecular mechanisms to pathophysiology (77), with the ultimate goal of improving the drug development process (78).

Mechanistic models have helped suggest the central role of DAMPs in acute inflammation (49, 75–76, 79–84). Mechanistic modeling has also helped elucidate the forces driving inflammatory preconditioning, namely the different inflammatory responses that ensue when multiple stimuli are given in succession (64, 85–90). Other applications of mechanistic modeling involve the understanding of multifactorial therapies for acute inflammatory diseases (91, 92). Key translational applications such as *in silico* clinical trials based on mechanistic models of inflammation and damage/dysfunction were pioneered in the arena of acute inflammation (71, 93, 94). These models have grown in sophistication, and are beginning to show the potential for predicting the inflammatory responses of large, outbred animals (78, 95, 96) and individual human subjects (71, 97, 98).

The unmet need for new treatments and diagnostic modalities allowing ultimately for long-term graft survival with low or no immunosuppression in organ transplantation is acute. While decades of work have led to many novel insights from the molecular to the physiological level, the net result has remained centered around life-long immunosuppression. We suggest that this is not because the effort has not been worthwhile or because promising candidate approaches were not pursued. Rather, it is our contention that what has not taken place is the process of synthesis of these insights into a larger whole. Computational modeling is a promising avenue for such synthesis; however, the current approach is based purely on statistical tools by which to associate multiple variables to outcomes.

In the present study, we created a mechanistic mathematical model based on ODEs that describe key mechanisms of innate and adaptive immunity and that span the full process of transplantation. This model focuses on the very early inflammatory events linked to the surgery, IRI, and memory T cell attack, events cross-modulated by each other and which translate into significant subclinical and clinical manifestations in only a subset of organ transplant recipients. However, these complex, early inflammatory events, as they do occur, may set the tone for either excellent or poor long-term allograft and patient outcomes. Thus, key outputs of our model include the prediction of that surgical injury and I/R-induced graft damage can be well-tolerated by the recipient when each is present alone, but that their combination (along with antigenic mismatch) may lead to acute rejection, as seen clinically in a subset of patients (38, 47). An emergent phenomenon from our simulations is that low-level DAMP release can tolerize the recipient to a mismatched allograft, whereas different restimulation regimens can drive an exaggerated rejection response. This former prediction is in agreement with published studies showing that preconditioning with the DAMP high-mobility group box 1 (HMGB1) can reduce the severity of inflammation and damage in the setting of graft IRI (99).

Limitations of this mechanistic mathematical model reside in the fact that the induction therapy and the maintenance immunosuppression are not considered in the model, and this is an area for expansion and augmentation of our modeling work. Moreover, this mechanistic mathematical model that predicts early innate and adaptive immune events is a generic one: each organ may have its own distinctive signature of early immune events. Thus, further augmentation of our model would involve making organ-specific variants. Additional limitations include the fact that this is a relatively abstract model, in which multiple mechanisms are lumped into single variables. As such, this model cannot be directly verified in a quantitative manner, other than as concerns the relative timing of various events. One key area where this limitation is apparent concerns the aforementioned emergent tolerization behavior as a function of prior exposure to damaged graft tissue, which we hypothesize as being due to DAMPs such as HMGB1 (99). Given tolerization is a manifestation of similar mechanisms to those that drive injury, and that HMGB1 can drive hepatic injury through activation of DCs (100), it is tempting to speculate that DCs are a key cell type in this process. Thus, future modeling work focused on examining this tolerization mechanism (or alternative mechanisms) in the context on organ-specific environments is warranted. In addition, a greater in-depth mathematical analysis can be done to gain deeper insights into the dynamics, which becomes especially helpful when the models are more closely tied to experimental and clinical data.

Despite these limitations, this model was capable of reproducing a rich set of biological and clinical behaviors. Simulations of this model under various initial conditions of IRI, graft injury, and degree of antigenic mismatch yielded a broad spectrum of outcomes from nearly complete graft function to outright (acute or chronic) rejection. Importantly, this model also yielded behaviors such as tolerization (durable unresponsiveness to donor-antigens) through preconditioning, as well as the harmful alternative outcome of more severe graft failure upon retransplantation. Future iterations of this model could address these limitations and additionally explore the effects of variability that would naturally exist from patient to patient with respect to host health and immune function (94). Consequently, mathematical/engineering control methodologies could be employed on the models to suggest early therapeutic intervention strategies for this complex immune system (101).

In conclusion, we suggest that this model is a stepping stone toward further insights, not only into the response to allotransplantation but also for other disease states. Several diseases with or without an immunologic trigger have been recently determined to have inflammation as a common fingerprint. Therefore, understanding diseases according to their common biological mechanism and using systems biology, mathematical modeling, and bioinformatics/data-driven modeling methods to interrogate the immune response before, during, and after perturbation will help not only to predict clinical outcomes but also guide prompt and precise targeting of new therapies (46, 102).

## Materials and Methods

We formulate the model by building upon the approach and principles of prior modeling work to provide the foundation for the current model (64, 69). In this prior work, an abstract, four-equation model of the acute inflammatory response to bacterial pathogen and to Gram-negative bacterial endotoxin was developed. The approach considered various subsystems as a way to tractably analyze and calibrate the qualitative behavior of parts of the larger system to gain a greater understanding of which entities governed certain dynamic properties in the larger system. We refer to this modeling process as a “subsystem modeling approach.” The Reynolds et al.’s model displayed rich qualitative behavior that corresponded to multiple clinical outcomes seen in cases of severe systemic inflammation due to bacterial pathogen and experimental studies of endotoxemia and tolerance. The general dynamical components of this prior model, when considered without a pathogenic or endotoxin insult, also correspond well to an abstract representation of the immune response to traumatic insult. Thus, the current model adopts a similar strategy and mindset for the development of the current model of immune responses in transplantation.

All model simulations and analysis were performed with XPPAUT (103). To create Figures 2–5, the numerical data produced from the XPPAUT simulations were exported to MATLAB^®^ (R2013b, The Mathworks Inc., Natick, MA, USA). Additional calculations were performed with MAPLE (2015, Maplesoft™, Waterloo, ON, Canada). The complete mathematical model given by the ODE system (1)–(6) was analyzed using the subsystems approach mentioned above wherein the dynamics of a few interacting variables are examined prior to combining the equations altogether. Parameter values used in this section can be found in Table 4. In the subsystems we discuss throughout this section, of most interest is the number and stability properties of equilibria and how these change with parameter value changes. Equilibria of a system of differential equations occur at the intersections of nullclines which are the equations resulting from setting each differential equation to zero and solving the resulting system of algebraic equations. The points that satisfy this are naturally the system states at which there is zero rate of change (e.g.,dxdt=0), indicating an equilibrium state or fixed point. The dynamics of the ODE system are organized around these special points. For a system of two variables, the nullclines are especially useful for a geometric analysis of the system states and to observe how the shapes and positions of the nullclines change with changes to parameters or functional forms of the equation terms. Small perturbations of the system away from an equilibrium that cause the system solutions to return to the equilibrium as *t*→∞ define a locally asymptotically stable (or simply *stable*) equilibrium. If, on the other hand, the perturbation causes solutions to move away from said equilibrium, then we call the equilibrium *unstable*. We only concern ourselves with biologically feasible equilibria which are those in the positive orthant. The variables of the system are necessarily formulated to remain positive for all time and all parameters are positive as well. For more details regarding the terminology and mathematical analysis used, consult for instance (104).

(1) $$\frac{\mathrm{dI}}{\mathrm{dt}}=\underset{\begin{array}{c} \text{See equation for R below; gain from activation of}\\ \text{resting/circulating innate components by host and graft}\\ \text{damage-induced DAMPs release and activated pro-inflammatory}\\ \text{innate and T cell components; inhibited by anti-inflammatory components, A} \end{array}}{\underset{︸}{\frac{s_{\text{ir}}R}{\mu_{\mathrm{ir}}+R}}}\underset{\begin{array}{c} \text{Loss due to}\\ \text{natural decay} \end{array}}{\underset{︸}{-\mu_{\text{i}}I}},$$

where R=kid(D+DG)+kiiI+kitpTP1+(Aa∞)2 (see Table 3);

(2) dDdt=kdi⋅f(I1+(Aa∞)2︸Inhibition by I-associatedanti-inflammatory componets, A)︸Gain from injurious effects of pro-inflammatoryinnate components on host tissue−μdD︸Loss due to tissuerepair/regeneration,

where $f(x)=\frac{x^{6}}{x^{6}+x_{\mathrm{di}}^{6}};$

(3) dDGdt=−μdDG︸Loss due to tissuerepair/regeneration+kdgigG⋅f(I1+(Aa∞)2︸Inhibition by I-associatedanti-inflammatory components, A)︸Gain from injurious effects of pro-inflammatoryinnate components on graft tissue+αkdgtpG f(TP1+(TAb∞)2︸Inhibition by anti-inflammatory T-cells)︸Gain from injurious effects on graft tissueby alloreactive memory T-cells,

(4) dAdt=−μaA︸Loss due tonatural decay+sa︸Source term​+kai(I+kaidD)/(1+(Aa∞)2)1+((I+kaidD)/(1+(Aa∞)2))︸Gain from production by innate pro-inflammatory componentsand host tissue damage with saturation and with self-regulating inhibition+kataTA,︸Gain from production byregulatory/anti-inflammatoryT-cells, TA

(5) dTPdt=ktpiT0I1+(TAb∞)2︸Gain from activation of T cells byinnate components, I, alone; inhibitedby regulatory/anti-inflammatory T cells, TA+αktpigT0I⋅G1+(TAb∞)2︸Gain from activation of T cells by alloreactiveinnate components, I⋅G; inhibited byregulatory/anti-inflammatory T cells, TA+α⋅ktpt0gT0TP⋅G1+(TAb∞)2︸Gain from activation of T cells byalloreactive activated T cells, TP⋅G;inhibited by regulatory/anti-inflammatory T cells, TA−μtpTP,︸Loss due tonatural decay

(6) $$\frac{{\text{dT}}_{\text{A}}}{\text{dt}}=\underset{\begin{array}{c} \text{Gain from activation of T cells by}\\ \text{innate components, I, in the presence of}\\ \text{anti-inflammaotry components, A} \end{array}}{\underset{︸}{k_{\text{taia}}T_{0}\cdot I\cdot A}}+\underset{\begin{array}{c} \text{Gain from activation of T cells by}\\ \text{alloreactive activated T regulatory/anti-}\\ \text{inflammatry T cells, TA}\cdot \text{G, with saturaltion} \end{array}}{\underset{︸}{\alpha \cdot k_{\text{tat0g}}T_{0}\cdot G\cdot \frac{T_{\text{A}}}{1+T_{\text{A}}}}}\underset{\begin{array}{c} \text{Loss due to}\\ \text{natural decay} \end{array}}{\underset{︸}{-\mu_{\text{ta}}T_{\text{A}}.}}$$

### *D*_Total_/*I* subsystem: Total damage and early innate components

We will first consider a subsystem that examines the dynamics of tissue damage and associated DAMP release with the early innate components of interest herein, as described in Table 1. In (69), it was shown that a similar subsystem involving damage and early pro-inflammatory phagocytes contained a stable healthy equilibrium as well as another stable equilibrium corresponding to elevated damage and elevated immune components. We build upon the structure developed there to construct our subsystem here and discuss the resulting analysis afterward. We note that the terms contained within the ODEs that we formulate are based on the principle of mass action kinetics. For instance, Table 5 provides the system of reactions involving the resting/circulating innate components, *I*_R_, and the activated innate components, I. Table 3 then provides the details on how we use a quasi-steady-state assumption to reduce the *I*_R_/*I* system to a single equation, based on the rapid nature of the activation process.

**Table 3:** **Auxiliary model variables**.

Auxiliary variables	Variable description, equation, and modeling explanation	
*I*_R_	Resting/Circulating population of *I* components, such as neutrophils and monocytes, from which the *I* population is activated. When the *I*_R_ population is activated into *I*, via DAMPs for example, we assume that the activation is rapid and employs a quasi-steady state assumption. (See Table 5 for reactions governing *I*_R_ and *I*.) The result is incorporated into the equations in which *I*_R_ appears. (arbitrary units: *I*_R_-units) $I_{R}=\frac{s_{ir}}{\mu_{ir}+k_{id}(D+D_{G})+k_{ii}I+k_{itp}T_{P}}$, derived from assuming that the following equation is in quasi-steady state: $\frac{{\text{dI}}_{\text{R}}}{\text{dt}}=s_{ir}-\mu_{ir}I_{R}-\left(k_{id}(D+D_{G})+k_{ii}I+k_{itp}T_{P}\right)I_{R}.$ In the equation for *I*, we let R=kid(D+DG)+kiiI+kitpTP1+(Aa∞)2 which incorporates the inhibitory effects of the anti-inflammatory mediators, represented in the variable, *A*, on the activation of *I*.	
*T*_0_	Population of inactivated memory T cells from which the T cell subsets, *T*_P_ and *T*_A_, are produced. The *T*_0_ population is also assumed to be in quasi-steady state and the result is incorporated into the equations in which *T*_0_ appears. (arbitrary units: *T*_0_-units) $T_{0}=\frac{s_{\text{t0}}(1+T_{\text{A}})}{\alpha k_{tat0g}T_{A}\cdot G+(\mu_{t\text{0}}+k_{tpi}I+\alpha k_{tpig}I\cdot G+k_{taia}A\cdot I+\alpha k_{tpt0g}T_{P}\cdot G)(1+T_{A})}$, derived from assuming that the following equation is in quasi-steady state: $\frac{dT_{0}}{dt}=s_{t0}-\mu_{t0}T_{0}-k_{tpi}I\cdot T_{0}-\alpha k_{tpig}T_{0}\cdot I\cdot G-\alpha k_{tpt0g}T_{0}\cdot T_{P}\cdot G-k_{tai}A\cdot I\cdot T_{0}-\alpha k_{tat0g}T_{0}\cdot G\frac{T_{A}}{1+T_{A}}$.	
*G*	Graft health/functionality; measured as a percentage with 0 indicating 0% functionality and 1 indicating 100% functionality. Graft health is defined as a function of associated graft damage, *D*_G_: $G=1-\frac{1-e^{\frac{{\text{-D}}_{\text{G}}}{{\text{x}}_{\text{gdg}}}}}{1+k_{\text{gdg}}e^{\frac{{\text{-D}}_{\text{G}}}{{\text{x}}_{\text{gdg}}}}}$. [Frame1]The parameters *k*_gdg_ and *x*_gdg_ scale the level of the variable *D*_G_ to relate it to the loss of graft functionality, denoted by the term$-\frac{1-e^{\frac{{\text{-D}}_{\text{G}}}{{\text{x}}_{\text{gdg}}}}}{1+k_{\text{gdg}}e^{\frac{{\text{-D}}_{\text{G}}}{{\text{x}}_{\text{gdg}}}}}$in the equation for *G*. See inset figure for an example response curve of *G*.	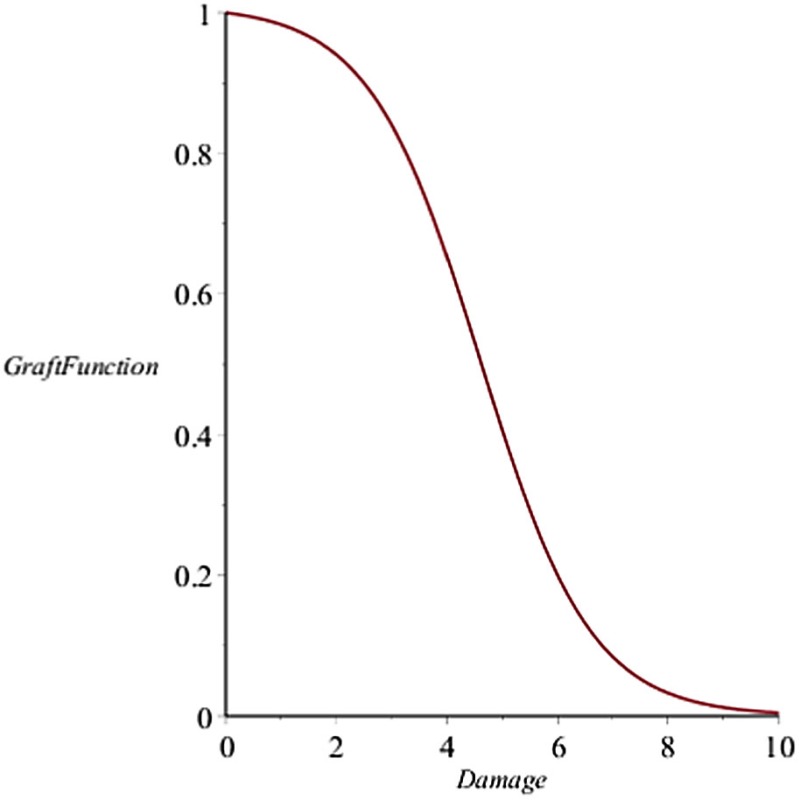

For the analysis of the *D*_Total_/*I* subsystem, we model the activation of resting/circulating pro-inflammatory innate components as described in Section “Deciphering the Complexity of Inflammation and Immunity with Mathematical Models” but ignore for now any inhibitory effects from anti-inflammatory components or additional activation by pro-inflammatory T cells and thus arrive at Eq. 7.

(7) $$\frac{\text{dI}}{\text{dt}}=\frac{s_{\text{ir}}(k_{\text{id}}D_{\text{Total}}+k_{\text{ii}}I)}{\mu_{\text{ir}}+(k_{\text{id}}D_{\text{Total}}+k_{\text{ii}}I)}-\mu_{\text{i}}I$$

(8) $$\frac{{\text{dD}}_{\text{Total}}}{\text{dt}}=\underset{\begin{array}{c} \text{Gain from effects}\\ \text{of early innate}\\ \text{components on}\\ \text{host tissue} \end{array}}{\underset{︸}{k_{\text{di}}\cdot f(I)}}-\underset{\begin{array}{c} \text{Loss from tissue}\\ \text{repair/regeneration} \end{array}}{\underset{︸}{\mu_{\text{d}}(D_{\text{Total}})}}+\underset{\begin{array}{c} \text{Gain from effects}\\ \text{of early innate}\\ \text{components on}\\ \text{graft tissue} \end{array}}{\underset{︸}{k_{\text{dig}}\cdot f(\text{I}\cdot \text{G})}},$$

where $f(x)=\frac{x^{6}}{x^{6}+x_{\mathrm{di}}^{6}}.$

The total tissue damage can be modeled by combining tissue injury caused (a) to host tissues from the early innate components responding to DAMP release and (b) to the graft, *G*, by either early innate components, *I*, or by pro-inflammatory T cells, *T*_P_, the latter of which is ignored for the analysis of the *D*_Total_/*I* subsystem. Thus, we formulate Eq. 8, where a decay term of the total damage is also incorporated to account for a combination of tissue repair and regeneration. Graft health, *G*, is a function of graft damage, *D*_G_ as discussed in Table 3. Note that since Eq. 8 is for total damage and not just graft damage, the parameters *k*_gdg_ and *x*_gdg_ have a slightly different meaning in this subsystem than they will in the full system, where the *D*_Total_ equation is separated into two equations: one to represent the damage to the host, *D*, and another to represent the damage to the graft, *D*_G_. This separation is done later in order to distinguish between damage done in general and graft-specific damage. Additionally, the inhibitory effects of anti-inflammatory components, *A* and *T*_A_, are later incorporated as is the additional damage to graft tissue by activated pro-inflammatory T cell subsets, *T*_P_.

As in (69), we assume that the ability of the innate immune components to create damage saturates when these components are very large relative to their baseline levels. We also incorporate the Hill-type function given as *f*(*x*) under Eq. 8 with a hill-coefficient of 6. We note that the choice of a hill coefficient in Reynolds et al. was made to ensure that the healthy equilibrium of the subsystem has a reasonable basin of attraction. Using the parameter values given in Table 4, this modified system behaves as in the prior work, with the *I* and *D*_Total_ nullclines intersecting at (0,0) and at two additional points in the positive quadrant. The “healthy equilibrium” (*D*_Total_, *I*) = (0,0) is locally asymptotically stable when $\mu_{\text{i}}>\frac{s_{\text{ir}}k_{\text{ii}}}{\mu_{\text{ir}}}$, which is the same criteria reached in (69) for the analogous parameters, even with the modifications made for this current focus. Furthermore, an unstable saddle equilibrium separates the basins of attraction of the healthy equilibrium and the other stable equilibrium (*D*_Total_, *I*)≈(1.2,17.5), as observed in the prior work. Thus, the underlying structure of bi-stability is present in the *D*_Total_/*I* subsystem we developed here. This means that the system has the ability to display different outcomes, depending on the initial conditions of the variables that we test. These outcomes are then translated qualitatively into clinical scenarios as discussed in Section “Results.”

**Table 4:** **Model parameters**.

Name	Description/source	Value/units	Name	Description/source	Value/units
*k*_ii_*	Activation of innate components by previously activated innate pro-inflammatory components	0.01/*I*-units/h	*S*_a_*	Source term for anti-inflammatory components (*A*)	0.0125 *A*-units/h
*S*_ir_*	Source of resting/circulating inactivated innate pro-inflammatory components	0.08 *I*_R_-units/h	*k*_ai_*	Maximum induction rate of anti-inflammatory components by activated pro-inflammatory innate components	0.04 *A*-units/h
*μ*_ir_*	Natural decay/turnover rate of resting/circulating inactivated pro-inflammatory innate components	0.12/h	*k*_aid_*	Relative effectiveness of activated pro-inflammatory innate components and damaged tissue/DAMPs to induce anti-inflammatory components (*A*)	48.0 *I*-units/*D*-units
*μ*_I_*	Natural decay/turnover rate of activated pro-inflammatory innate components	0.05/h	*μ*_a_*	Decay rate of anti-inflammatory components (*A*)	0.1/h
*k*_id_*	Activation of resting/circulating pro-inflammatory innate components by DAMP release from damaged host and graft tissue	0.02/(*D-D*_G)_-units/h	*k*_ata_	Maximum induction rate of anti-inflammatory components (*A*) by anti-inflammatory T cells (*T*_A_)	0.001/*A*-units/*T*_A_*-*units/h
*k*_itp_	Activation of resting/circulating pro-inflammatory innate components by activated/memory pro-inflammatory T cells	0.008/*T*_P_-units/h	*S*_t0_	Source of inactivated memory T cells	1.0 *T*_0_-units/h
*k*_di_*	Maximum rate of host tissue damage by activated pro-inflammatory innate components	0.35 *D*-units/h	*μ*_t0_	Decay rate of inactivated memory T cells, *T*_0_.	0.05/h
*x*_di_*	Determines level of activated pro-inflammatory innate components that increases damage production to half its max	0.06 *I*-units	*k*_tpi_	Maximum activation rate of memory T cells by pro-inflammatory innate components	0.008/*I*-units/h
*k*_dgig_	Maximum rate of graft tissue damage by activated pro-inflammatory innate components	0.35*D*_G_-units/h	*k*_tpig_	Maximum activation rate of memory T cells by pro-inflammatory innate components in the presence of allo-Ag	0.02/*I*-units/h
*μ*_d_*	Decay rate of host and of graft tissue damage representing repair/regeneration of injured tissue	0.02/h	*k*_tpt0g_	Maximum activation rate of memory T cells by alloreactive activated memory T cells	0.02/h
α	Scaling parameter that governs the level of apparent mismatch between host and graft α = 0 indicates 0% mismatch; α = 1 indicates 100% mismatch	α∈[0,1] dimension-less	*μ*_tp_	Decay rate of activated pro-inflammatory memory T cells	0.03/h
*k*_dgtp_	Maximum rate of damage by pro-inflammatory T cells to graft tissue scaled by the parameter α; set to be greater than *k*_id_ and *k*_dgig_ to indicate greater potency of alloreactive T cells	0.7 *D*_G_-units/h	*k*_taia_	Maximum induction of anti-inflammatory T cells by pro- and anti-inflammatory innate components	0.04/*I*-units/*A*-units/h
*x*_dgtp_	Determines the level of pro-inflammatory T cells that increases graft tissue damage to half its max	1 *D*_G_-units	*k*_tat0g_	Maximum activation rate of anti-inflammatory T cells by already activated alloreactive anti-inflammatory T cells	0.001/h
*k*_gdg_	Tuning parameter that governs the response curve of the graft function, G (See Table 3)	10 dimension-less	*μ*_ta_	Decay rate of activated anti-inflammatory T cells	0.03/h
*x*_gdg_	Tuning parameter that governs the response curve of the graft function, G (See Table 3)	0.5 *D*_G_-units	*b*_∞_	Controls the strength at which the anti-inflammatory T cells (T_A_) inhibit various processes	0.5 T_A_-units
*a*_∞_*	Controls the strength at which the anti-inflammatory components (*A*) inhibit various processes	0.28 *A*-units			

*Parameters marked with an asterisk retain the baseline value as set in (69)*.

**Table 5:** **Reactions involved in the *I*_R_*/*I subsystem**.

$I_{\text{R}}\overset{k_{\text{di}}(D+D_{\text{G}})+k_{\text{ii}}I+k_{\text{itp}}T_{\text{P}}}{\to }I$	Activation of resting/circulating innate components, *I*_R_, by damaged host tissue, *D*, damaged graft tissue, *D*_G_, activated innate cells or mediators, *I*, and pro-inflammatory T cells, *T*_P_
$*\overset{s_{\text{ir}}}{\to }I_{\text{R}}$	Source of resting/circulating innate components, *I*_R_
$I_{\text{R}}\overset{\mu_{\text{ir}}}{\to }$	Natural decay of resting/circulating innate components, *I*_R_
$I\overset{\mu_{\text{i}}}{\to }$	Natural decay of activated innate components, *I*

Additionally, we know from the prior results that the incorporation of the anti-inflammatory component when treated as a constant will yield a loss of this bi-stability when the level of the anti-inflammatory component exceeds a value of 0.6264 and only the healthy equilibrium remains stable. Therefore, when we incorporate the analogous dynamic anti-inflammatory component, *A*, into the full model, we wish to make sure to calibrate any additions to *A* such that the maximum level of *A* does not exceed the 0.6264 threshold, since this would produce unreasonable (i.e., non-biological) behavior. For instance, if this threshold were exceeded, the *D*_Total_/*I* subsystem would be incapable of reaching an unhealthy equilibrium while other components of the model, such as activated pro-inflammatory T cells or graft damage (when separated from total damage), would remain elevated. The conditions for bi-stability noted above will not be changed when we combine subsystems at the end.

### The *I*/*T*_P_ subsystem

(9) $$\frac{\text{dI}}{\text{dt}}=\frac{s_{\text{ir}}k_{\text{ii}}I}{\mu_{\text{ir}}+k_{\text{ii}}I}-\mu_{\text{i}}I$$

(10) $$\frac{{\text{dT}}_{\text{P}}}{\text{dt}}=k_{\text{tpi}}T_{0}\cdot I-\mu_{\text{tp}}T_{\text{P}},$$

The *I*/*T*_P_ system has one or two non-negative equilibria depending on the parameter values. If we fix the values for the parameters that appeared in the *D*_Total_/*I* system, the following parameters govern the number and stability of the equilibria: *k*_itp_, *s*_t0_, μ_t0_, *k*_tpi._ The point (*I*, *T*_P_) = (0,0) is always an equilibrium and is stable for *k*_itp_ = 0.01, *s*_t0_ = 1, μ_t0_ = 0.05, and *k*_tpi_ = 0.01. Since we have an estimate for the half-life of activated T cells (unpublished work) which translates to a rate of 0.03/h, we estimate the half-life of inactivated memory T cells to be slightly longer than this at 0.05/h. Also we fix the source term, *s*_t0_, to a value of 1 and then determine the values of *k*_itp_ and *k*_tpi_ such that the (0,0) equilibrium is stable and that the rate at which trajectories approach this equilibrium is not unduly slow, which is related to the position of the nullclines. For simplicity, we let *k*_itp=_*k*_tpi_ since there is a lack of information regarding the relative strength at which one incites the other. Setting the value of *k*_itp_ = 0.008 = *k*_tpi_ allows for each variable to contribute to recruiting the other by a non-negligible amount in this subsystem and are, as a pair of values, not too close to a bifurcation value where the nullclines would cross a second time. In other words, if their values are set to 0.01, for example, then (0,0) will be unstable; however, we wish for this subsystem to have (0,0) stable under these parameters so that neither *I* nor *T*_P_ will drive sustained *T*_P_ or *I* levels, respectively. Therefore, when connected to the damage equations, when each is sustained at an elevated equilibrium, this will depend on feedback from the damage they incite, rather than just each other.

### The *D*_G_/*T*_P_ (and *G*) subsystem

(11) $$\frac{{\text{dD}}_{\text{G}}}{\text{dt}}=-\mu_{\text{d}}D_{\text{G}}+\alpha k_{\text{dgtp}}G\cdot f(T_{\text{P}}),$$

where $f(x)=\frac{x^{6}}{x^{6}+x_{\mathrm{di}}^{6}};$

(12) $$\frac{{\text{dT}}_{\text{P}}}{\text{dt}}=\alpha \cdot k_{\text{tpt0g}}T_{0}\cdot T_{\text{P}}\cdot G-\mu_{\text{tp}}T_{\text{P}}.$$

For the *D*_G_/*T*_P_ subsystem that includes the auxiliary variable *G*, the same type of functional form used in modeling damage to host (*D*) via innate cells (*I*) is employed to model the graft damage, *D*_G_, created by pro-inflammatory T cells, *T*_P_. The parameter values are set according to Table 4. Bi-stability is not a feature of this system, but when there are no *T*_P_ T cells, then (0,0) is always stable; and for low mismatch factor (i.e., α ≤ 0.074), (0,0) is stable. As α increases through this, (0,0) becomes unstable and a new equilibrium of interest is born and is stable (spiral). For values close to 0.075, the approach to the equilibrium is quite slow away from the stable manifolds of the equilibrium. When α = 0.08, the positive equilibrium is a stable spiral which establishes the presence of damped oscillations in this subsystem. Naturally, as T cells destroy graft tissue, there is less tissue to destroy, but as the tissue regenerates, the T cells can then destroy this regenerated tissue. Also, as T cell numbers increase the source for new ones is depleted until the turnover/death of existing activated T cell subsets allow for the activation of more (literally the way the source/recruitment term is modeled) – this could be interpreted as a wait time for replenishment of the T cell source from the bone marrow. Understanding the tissue repair process and time scale relative to T cell behavior could help calibrate this aspect better. For instance, tissue repair/regeneration may be hindered significantly in disease states and therefore may depend on the existing level of damaged tissue.

### The *D*_G_/*T*_P_/I (and G) subsystem

The *D*_G_/*T*_P_/I subsystem which includes the auxiliary variable *G* is given by Eqs 13 and 14 and displays bi-stability for the parameters listed in Table 4 (with α = 0). Note that the *D*_Total_\*I* subsystem is partially contained in this 3-variable subsystem. Initial graft damage values in which *D*_G_(0) > 0.095 lead to graft/host failure. Recall that this behavior is in the absence of any anti-inflammatory inhibition; so very little graft damage can lead to failure in this subsystem even without a positive mismatch factor. For very low initial graft damage [e.g., *D*_G_(0) = 0.08 or ~2% graft damage] and for low graft mismatch (e.g., α = 0.01), survival is possible. Though the ranges of pairs of values of initial graft damage and α that produce survival outcomes is limited, the presence of bi-stability exists and the presence of inhibitory components added later allow this range to increase. For some *D*_G_(0) and α value pairs [e.g., *D*_G_(0) = 0.08 and α = 0.02], graft functionality remains very high (~99%) for ~230 h (~1 week) after which it decreases rapidly to its ending steady-state functionality value of 13% by ~300 h.

If considering the presence of activated memory T cells at time zero [i.e., *T*_P_(0) > 0], the time to graph failure greatly decreases. For example with *D*_G_(0) = 0.08 and α = 0.02, when *T*_P_(0) = 1 functionality decreases to 13% by 50 vs. 300 h without an initial population of activated memory T cells. A similar result occurs when there is an initial population of activated innate inflammatory components, *I*. For example, with *D*_G_(0) = 0.08, α = 0.02, and *T*_P_(0) = 0, when *I*(0) = 0.01, graft functionality decreases to 13% by 165 vs. 300 h without an initial population of activated innate inflammatory components.

(13) $$\frac{\text{dI}}{\text{dt}}=\frac{s_{\text{ir}}R_{1}}{\mu_{\text{ir}}+R_{1}}-\mu_{\text{i}}I,$$

where $R_{1}=k_{\text{id}}D_{\text{G}}+k_{\text{ii}}I+k_{\text{itp}}T_{\text{P}};$

(14) $$\frac{{\text{dD}}_{\text{G}}}{\text{dt}}=-\mu_{\text{d}}D_{\text{G}}+k_{\text{dgig}}G\cdot f(I)+\alpha k_{\text{dgtp}}G\text{​}f(T_{\text{P}}),$$

where $f(x)=\frac{x^{6}}{x^{6}+x_{\mathrm{di}}^{6}};$

(15) $$\frac{{\text{dT}}_{\text{P}}}{\text{dt}}=k_{\text{tpi}}T_{0}\cdot I+\alpha k_{\text{tpig}}T_{0}\cdot I\cdot G+\alpha k_{\text{tpt0g}}T_{0}\cdot T_{\text{P}}\cdot G-\mu_{\text{tp}}T_{\text{P}}.$$

### Anti-inflammatory effects

The parameter values for the anti-inflammatory components, *A*, were set as in Reynolds et al. where applicable and the additional parameters in this category were estimated to calibrate the baseline responses. For instance, the contribution of *T*_A_ to *A* was calibrated such that maximum *T*_A_ levels would not allow *A* to exceed its threshold value of 0.6264 as discussed previously. Additionally, in the case of severe initial tissue damage, it is possible that this positive feedback between DAMP release caused by tissue injury and inflammation causing further tissue injury may not resolve, and thus lead the way to multi organ failure and death. In the current state-of-the-art, the transplantation procedure and donor graft condition are such that the surgical procedure and associated I/R are typically not the cause of organ failure. However, theoretically, this scenario is possible and helps to calibrate the extreme cases of the model such that complete resolution is not the only outcome possible regardless of initial conditions and parameter values. Thus, the inhibitory effects of *A* and *T*_A_ combined do not overly and unrealistically dampen the inflammatory arm of the responses. We retain the positive background level of the anti-inflammatory component at the non-perturbed healthy equilibrium, *A*, as set in Reynolds et al.: *A*(0) = *A*_0_ = 0.125 (69).

## Conflict of Interest Statement

Yoram Vodovotz is a co-founder and stakeholder in Immunetrics, Inc. This conflict is being managed by the University of Pittsburgh under all pertinent institutional and federal regulations. The remaining co-authors declare that the research was conducted in the absence of any commercial or financial relationships that could be construed as a potential conflict of interest.

## Footnotes

^1^http://optn.transplant.hrsa.gov/
